# Environment as a Potential Key Determinant of the Continued Increase of Prostate Cancer Incidence in Martinique

**DOI:** 10.1155/2011/819010

**Published:** 2011-11-30

**Authors:** Dominique Belpomme, Philippe Irigaray

**Affiliations:** ^1^Paris Descartes University, 75015 Paris, France; ^2^Clinical Cancer Research Department, European Cancer and Environment Research Institute (ECERI), 1000 Bruxelles, Belgium; ^3^Association for Research and Treatments Against Cancer (ARTAC), 75015 Paris, France

## Abstract

Prostate cancer incidence is steadily increasing in many developed countries. Because insular populations present unique ethnic, geographical, and environmental characteristics, we analyzed the evolution of prostate cancer age-adjusted world standardized incidence rates in Martinique in comparison with that of metropolitan France. We also compared prostate cancer incidence rates, and lifestyle-related and socioeconomic markers such as life expectancy, dietary energy, and fat supply and consumption, with those in other Caribbean islands, France, UK, Sweden, and USA. The incidence rate of prostate cancer in Martinique is one of the highest reported worldwide; it is continuously growing since 1985 in an exponential mode, and despite a similar screening detection process and lifestyle-related behaviour, it is constantly at a higher level than in metropolitan France. However, Caribbean populations that are genetically close to that of Martinique have generally much lower incidence of prostate cancer. We found no correlation between prostate cancer incidence rates, life expectancy, and diet westernization. Since the Caribbean African descent-associated genetic susceptibility factor would have remained constant during the 1980–2005, we suggest that in Martinique some environmental change including the intensive use of carcinogenic organochlorine pesticides might have occurred as key determinant of the persisting highly growing incidence of prostate cancer.

## 1. Introduction

Prostate cancer incidence is steadily increasing in many developed countries, where it is commonly attributed to improvement in screening detection and to population ageing [1]. We have previously analysed these two factors [2], and in response to a recent article [3], we have argued that overdiagnosis by the routine use of prostate-specific antigen (PSA) test cannot fully account for the growing incidence of this cancer [4]. Furthermore, increase in life expectancy does not explain why overall the rise of cancer incidence affects all age categories [5] and why it occurs earlier in life [6].

In a previous multifactorial study, we have suggested that in the two French Caribbean islands, Martinique and Guadeloupe, prostate cancer may in fact be caused by environmental factors and that among these factors, carcinogenic organochlorine pesticides may play a role [7].

In this paper, we further attempt to show that in Martinique, environmental change may account for the growing incidence of prostate cancer in highly susceptible people and discuss the role of exogenous carcinogens that may be involved.

## 2. Material and Methods

Because insular populations present unique ethnic, geographical, and environmental characteristics that may be well conserved, studies of populations of the Caribbean can help elucidate the aetiology of prostate cancer. We have chosen the tropical island Martinique, in the French West Indies, because of its limited territory (1128 km²), its low number of inhabitants (414 516), a medical practice and lifestyle-related behaviour that does not differ from metropolitan France, the availability of a cancer registry rigorously collecting and reporting cases, and the possibility of determining environment- and lifestyle-related factors and their time-related modifications.

In this ecological study, we have analysed the evolution of prostate cancer incidence rates in Martinique in comparison with that in metropolitan France during the period 1980–2005 and have compared the incidence rates obtained in 2005 with those of other Caribbean islands and of UK, Sweden, and USA. Data collection was done as follows: for Martinique, we used data from the Martinique cancer registry held by AMREC, the Martinique Association for Epidemiological Research on Cancer [8]. For comparison with metropolitan France, we used data from the French National Sanitary Surveillance Institute (InVS) [9], which provides incidence rates from 11 metropolitan “department” registries. These registries are those from which the national extrapolated incidence rates of prostate cancer in metropolitan France are based on. For international comparison, we used incidence rates from the Globocan 2008 database of the international Agency for Research on Cancer (IARC) [10]. However, since these data may have been highly extrapolated, we also used for comparison data collected by specific registries including the one of the public health ministry of Cuba [11], for UK, that is, for England, Scotland, and Wales, those from the Office for National Statistics [12], the Information Services Division, Scotland [13], and the Welsh Cancer Intelligence and Surveillance Unit [14], and for Sweden, USA, and metropolitan France, those from the National Board of Health and Welfare [15], the National Cancer Institute's Surveillance, Epidemiology and End Results (SEER) [16], and the InVS [9], respectively. Finally, in order to make data comparison, we only considered incidence rates that had been age-adjusted to the IARC world standard and expressed as age-standardized rates (ASR). Since in Martinique PSA screening does not differ from metropolitan France, data processing consisted of comparing the evolution of prostate cancer incidence rates in Martinique with that of metropolitan France. Furthermore, in order to determine the best model fitting incidence growth curves, we checked for growth homogeneity for each of the 11 metropolitan French “department” registries and for the registry of Martinique. For modeling, we used a least-square regression analysis and established curve equations according to the best values obtained for the determination coefficient *R*². Since the best model fits in exponential functions, data were linearized by log transformation. For comparison of the two groups, the interaction between group and time was analyzed by a mixed linear model, assuming an unstructured covariance matrix for the random effects and a first-order autoregression covariance structure for the within population correlation. Slopes were treated as random effect, thus the intercept at year 1985 is interpretable as initiation of growth, and the slope is interpretable as rate of growth for each population. Mathematical treatments were done using contrasts of fixed effects for the group slopes with inference based on the *F*-test. Estimation by restricted maximum likelihood (REML) was computed using SPSS v.16.0 (SPSS, Inc., Chicago, Ill, USA), and model suitability was assessed by Akaike's information criterion. Coefficients, confidence intervals (CI) of coefficients, and two-sided *P* values are reported for the model. Since it has been shown that due to some ethnographic genetic factor, there is a marked increase in prostate cancer incidence in African descents and because Caribbean people are African descents, for international comparison, we took into account the percentage of African descents in Caribbean, UK, and Sweden in the Encyclopedia of the Nations [17], the Office by National Statistics [18], and the Befolkningsstatistik [19], respectively. Unfortunately, due to legal regulation, data were not available for metropolitan France, but a common estimation is that this percentage is low and supposed to be not different from the percentage in UK and Sweden. We also considered a report from the French ministry of health indicating that the health care system in Martinique and Guadeloupe does not differ to that in metropolitan France [20]. In addition, we used several usually accepted socioeconomic markers of lifestyle-related behaviour, such as life expectancy at birth and food supply and consumption in order to make comparison. For comparing life expectancy at birth, we used data source from the WHO Core Health Indicators database for 2006 [21], and for comparing dietary energy and fat supply and dietary energy and fat consumption, we used data source from FAO Food Balance Sheets 1988–1990 [22] and data source from FAO Statistical Yearbook 2009 [23], respectively. We also used data from Eurostat [24] and from US-EPA [25] for pesticide use and exposure in the different countries or territories analyzed for which specific incidence registries were available. For Martinique, we used the determination we had previously made [7]. For determination of the correlation coefficient, *r*, we used the Spearman test.

## 3. Results

**Figure 1:**
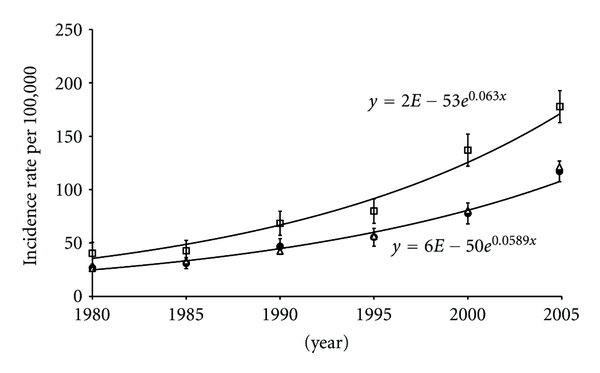
Evolution of prostate cancer incidence rates expressed as ASRs in Martinique □ in comparison with the incidence growth curve obtained from the 11 “department” registries of metropolitan France ● and with the extrapolated overall incidence growth curve for metropolitan France ∆. Values of *R*² were 0.9742 for Martinique and 0.9845 for the 11 metropolitan “department” registries. Note that for Martinique and metropolitan France, despite the fact they are seemingly diverging since 1985, after log transformation, the 2 curves are not significantly diverging (see Figure 2).

**Figure 2:**
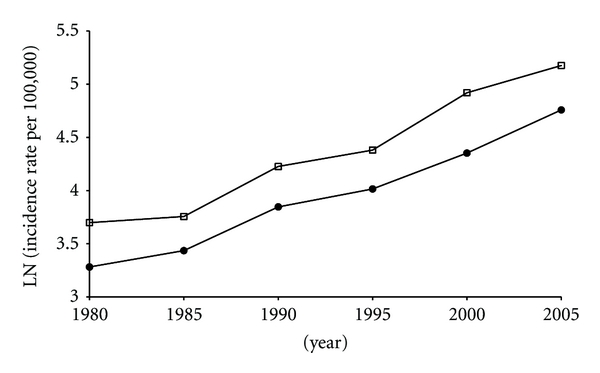
Evolution of Log transformed prostate cancer incidence rates expressed as ASRs for Martinique and metropolitan France. Incidence rates in Martinique are continuously at a higher level than in metropolitan France (*P* < 0.001).

Tables 1 and 2 and Figures 1 and 2 summarize our data. As indicated in Table 1, the world age-standardized incidence rate of prostate cancer in 2005 in Martinique is one of the highest worldwide whatever it has been determined from the Martinique specific registry of AMREC or estimated from the IARC Globocan 2008 database: 177 per 100 000 according to the AMREC registry and 173.7 per 100.000 according to the IARC Globocan database. This incidence rate is indeed higher than those obtained from specific registries for metropolitan France, Sweden, and USA and much higher than the ones reported for UK. However, surprisingly, despite the fact that with the exception of Cuba and Trinidad and Tobago, 80 to 95 percents of the Caribbean population are of African origin, as it is the case in Martinique, this incidence rate was found to be much higher than those reported by IARC in the Globocan 2008 database for Guadeloupe and other Caribbean islands and even higher than the one reported in 2003–2007 for African descents living in the USA.

 The growth curves of prostate cancer incidence rates expressed as ASRs during the period 1980–2005 (i.e., during one generation), respectively, for Martinique, for the 11 metropolitan “department” registries and for overall metropolitan France are displayed Figures 1 and 2. We found that the overall growth rate of incidence in Martinique as well as in metropolitan France is constant. Evaluation of the correlation between incidence ASRs and time confirmed indeed that both incidence growth curves fit in well an exponential function: mean *r* = 0.993 for Martinique and mean *r* = 0.990 for metropolitan France, with incidence growth curve equations in the form of *y* = 2*E* − 53*e* ^0.063*x*^ and of *y* = 6*E* − 50*e* ^0.0589*x*^ for Martinique and metropolitan France, respectively. No significant difference could be detected in the interaction of time by incidence rates for Martinique compared to metropolitan France (*F*_1,18.2_ = 0.68, *P* = 0.4 ). In other words, after log transformation, when compared to metropolitan France the overall growth rate of incidence of prostate cancer for Martinique is not significantly different (*β* = −0.004, *P* = 0.4, 95%, CI − 0.013 to 0.006). However, as displayed in Figure 2, the incidence rates for Martinique are significantly at a constant higher level than those for metropolitan France (0.416, *P* < 0.001  , 95%  *CI*  0.294  *to*  0.539). Table 1 also indicates that life expectancy at birth in Martinique and Guadeloupe is similar to that in France, UK, Sweden, and USA. By contrast, with the exception of Cuba, life expectancy at birth in the Caribbean islands other than Martinique and Guadeloupe is generally lower, in the range of 59 to 72 years of age. We found no correlation between prostate cancer incidence rates and life expectancy at birth (*r* = 0.239, *P* = 0.4), and similarly, as far as diet westernization is concerned, no correlation between prostate cancer incidence rates as determined by Globocan 2008 and dietary energy (expressed in calories) and fat consumption as determined by FAO (for calories: *r* = 0.235, *P* = 0.4, for lipids: *r* = 0.4, *P* = 0.1). However, when analyzing the pool of all countries or territories included in the study (see Table 1), we found a strong correlation between life expectancy and dietary energy and fat intake (*r* = 0.911, *P* = 0.001). Moreover, as suggested in Table 2, except for Sweden for which factors other than pesticides should be considered, we found some degree of correlation between the incidence rate of prostate cancer and the level of pesticide use in the different countries and territories analyzed, the higher the level is, the higher the prostate cancer incidence tends to be (*R*² = 0.67, *P* = 0.04).

## 4. Discussion

Despite the fact that prostate cancer is the most frequent diagnosed cancer and the second cause of cancer death in men in Western countries, its aetiology remains unclear. The only established risk factors are advancing age, family history, and ethnic origin [26]. However, risk factors are not necessarily cancer causing agents, that is, agents directly involved in the carcinogenesis process, but most often familial factors that contribute to genetic susceptibility and/or lifestyle-related factors that contribute to exposure to carcinogens and/or cocarcinogens [27]. Moreover, although environmental causes of prostate carcinogenesis have not yet been clearly established [26, 28], prostate cancer, as other cancers, is believed to result from a multifactorial process involving both genetic and environmental components [29, 30].

A major finding in the present study is that in Martinique, the incidence rate of prostate cancer is presently one of the highest reported worldwide (e.g., even higher than the one for the black people living in USA) and that it is continuously growing since 1985 in an exponential mode, at a growth rate not differing from that of metropolitan France (i.e., the initial difference remains constant) but that it is constantly at a higher level that differs significantly from that of metropolitan France, meaning that after log transformation, the two incidence growth curves are parallel. A similar trend in the continuously growing incidence rate of prostate cancer is reported in several countries in Europe, including Denmark, Finland, Norway, Sweden, Ireland, and The Netherlands [10, 31]. However, this trend is not observed in the USA, since after the prostate cancer incidence rate peaked in 1992, there is in this country for still undetermined reasons a decrease in prostate cancer incidence although the incidence rate in 2007 remains at a higher level than it was in 1975 [32].

In response to a recent published study carried out in the USA concerning prostate cancer diagnosis and treatment after the introduction of PSA screening [3], we have already discussed the fact that the introduction two decades ago of PSA-based screening techniques cannot explain the persisting growing incidence of prostate cancer in many developed countries [4]. Indeed, exponentially growing incidence rates, such as those reported in Figures 1 and 2 with no visible inflexion, tend to confirm our previous hypothesis according to which, in addition to screening, other factors should be considered, accounting for the continuously growing incidence [4]. Moreover, it has been clearly shown in several European countries that the rise in prostate cancer incidence started long before the initial use of PSA screening test [2, 32]. Unfortunately, there is no available data comparing the rate of use of PSA screening test per inhabitant in Martinique and metropolitan France. However, the health care system in Martinique is rigorously the same as it is in metropolitan France as far as organization, health expenditure, and training of physicians are concerned [20] and the date of PSA screening technique introduction has been identical in both cases. Consequently, it is unlikely that the significantly different higher level of incidence rates observed in Martinique might be due to a difference in screening. Indeed, if we suppose that during our study observation period, the incidence of prostate cancer observed in Martinique, which is situated far away from metropolitan France, would have been associated with a less frequent use of PSA test, the results would have been exactly the opposite of what we observed, that is, a lower rate of prostate cancer incidence. Inversely, for similar reasons, it would be not meaningful to speculate that a less frequent use of PSA test would account for the lower incidence rate of prostate cancer observed in metropolitan France, since the PSA test has been initially developed in this country.

Similarly, life expectancy at birth of the population in Martinique does not differ from the one in metropolitan France (Table 1), confirming that quality of health care system, socioeconomic status, and lifestyle-related behaviour of people living in Martinique and metropolitan France cannot *per se* account for the observed difference in incidence. Therefore, this led us to look for other parameters which could account for the higher incidence rate of prostate cancer in Martinique as compared to metropolitan France.

As observed in USA, men of African descents when compared to Caucasians have been shown to be associated with an ethnographic genetic factor making them more susceptible to prostate carcinogenesis while they both are living in the same environment [33]. Therefore, the difference in incidence rates between Martinique and metropolitan France could be explained from a genetic perspective by the African origin of Caribbean population [34]. Considering the incidence growth curve in Martinique is constantly at a significantly higher level than it is in metropolitan France, and that after log transformation this growth curve is parallel to that of metropolitan France (see Figures 1 and 2), this strongly suggests that not only a Caribbean African descent-associated genetic susceptibility factor is involved in prostate carcinogenesis in Martinique, as it is the case for American African descents living in the USA [35], but also that this factor remained constant during the one generation observation period (1980–2005). However, the local environment in Martinique and metropolitan France is quite different. As indicated in Table 1, albeit they are genetically close if not equivalent to that of Martinique and living in similar regional areas Caribbean populations appear generally to have much lower prostate cancer incidence rates. This suggests that in addition to the ethnographic genetic factor, a nongenetic factor or rather a strong interaction between genetic and environmental factors may be involved in countries or territories with high rates of prostate cancer incidence. However, values of prostate cancer incidence in Caribbean countries or territories where there is no available specific cancer incidence registry may be underestimated, because uptake of PSA testing might be lower, as it may be the case in USA for black men in comparison to with Caucasians [36]. As discussed above, a difference in PSA screening use between Martinique and metropolitan France is unlikely. Furthermore, as reported in Table 1, Cuba for which a specific cancer incidence registry does exist is associated with a significant lower prostate cancer incidence rate than in the USA despite the fact there is a similar percentage of African descents in both countries. Yet, a similar discrepancy does exist when comparing the prostate cancer incidence rate in Sweden to that in UK, while these countries, which both have similar high level health care systems and excellent specific cancer incidence registries, have a similar percentage of African descents (Table 1). With regards to Martinique and metropolitan France, it would have been instructive to know the incidence rate of prostate cancer in the Caucasian population in Martinique. Unfortunately, such data are not available. As reported by IARC in the Globocan 2008 database, the discrepancy between the incidence rates in Martinique and Guadeloupe should be noted considering that the population and local environment are seemingly similar if not identical. Therefore, it appears that an environmental factor specific to Martinique could be responsible for the higher elevated prostate cancer incidence rate in this island.

On the basis of epidemiological studies, an increase in prostate cancer incidence in people migrating from low cancer incidence countries to high incidence ones [35, 37, 38] has been observed, suggesting that lifestyle-related and/or environmental factors could be potential risk factors for prostate cancer [39, 40]. However, the carcinogenic role of so-called westernized dietary regimens which mainly consists of a low intake of antioxidants still remains unclear. The association of prostate cancer risk with dietary factors such as high intake of fat, meat, and dairy products has been considered [35, 41], but several epidemiological studies have shown conflicting negative results [35, 42]. On the basis of our analysis of international available data, we found that life expectancy at birth was strongly correlated with dietary energy and fat supply or consumption, whereas we could not find any correlation between prostate cancer incidence and dietary energy and fat supply or consumption. For example, despite the fact that during the period 1988–1990, Cuba, was believed to have one of the highest level of daily calories per person in the Caribbean, as indicated in Table 1, prostate cancer incidence is the lowest, whereas albeit Martinique had the lowest level of daily calories per person in comparison with the ones in UK, Sweden, France, and USA, and for this reason is considered to be associated with a modest diet westernization [43], prostate cancer incidence is the highest. A further argument suggesting a possible role of environmental causes in the growing incidence of prostate cancer is that although UK is associated with a high level of dietary energy and fat supply and consumption similar to that in USA, Sweden, and France, prostate cancer incidence rate is one of the lowest of Western countries, as it is the case for Cuba (Table 1). And this is particularly true for men of African or Caribbean origin living in UK, since for this specific population, prostate cancer incidence rate is 70% less than the corresponding one for African descents living in USA [34]. Moreover, it has been shown in the European prospective investigation into cancer and nutrition (EPIC) study that fruits and vegetables do not protect against prostate cancer [44]. These data therefore strongly support the concept that risk factors other than those related to lifestyle are associated with prostate cancer occurrence, that dietary antioxidants do not play a protective role against prostate cancer, and consequently that mechanisms other than free radicals production are involved in prostate carcinogenesis [30].

Lifetime exposure to endogenous androgens and estrogens has been suggested to be a risk factor for prostate cancer [45, 46], but this endogenous model does not fit in the results of the present study showing a continued increase of cancer incidence since 1985.

We have previously distinguished lifestyle-related risk factors from environmental cancer-causing agents and defined the latter as exogenous physical, chemical, and biological carcinogens or cocarcinogens [2, 4, 47].

As shown in Figures 1 and 2, although significantly differing in levels, the two incidence rate growth curves follow a similar exponential pattern. This may reflect a similar overall effect of different environmental factors, in the framework of gene-environment interactions, whatever these factors could be. In many developed countries including metropolitan France, such factors are unknown. The lack of major industries and associated sources of industrial pollution in Martinique suggests that a factor linked to agriculture may be involved, considering that agriculture is the main economic activity of the island. As indicated in Table 3, several carcinogenic, mutagenic, and/or reprotoxic (CMR) or presumed CMR pesticides including dichloro-diphenyl-trichloroethane (DDT), hexachlorocyclohexane (HCH), chlordanes, aldrin, dieldrin, chlordecone, and simazine have been used in great quantities since 1950 in Martinique for the preventive treatment of banana plantations. We have shown that several of these pesticides used between 1950 and 1970 in Martinique have been detected at considerably high levels in the adipose tissue of all subjects tested [7]. In Martinique, as it is the case for prostate cancer, there is also a recently growing incidence of breast carcinoma [8], and we have proposed that organochlorine pesticides alone or through cocktail effects could cause both prostate and breast cancers by acting through similar common endocrine disruption mechanisms [48]. Many epidemiological studies—but not all—have reported that exposure to organochlorine pesticides is associated with an increased risk of prostate cancer and that among the different pesticides which have been used intensively since 1950 in Martinique, DDT and 1,1-dichloro-2,2′-bis-p-chlorophenyl-ethylene (DDE) [49, 50], Lindane [51], aldrin and dieldrin [49], chlordane [49], heptachlor [49, 51], oxychlordane [52, 53], and the nonorganochlorinated pesticide simazine [53] are associated with a significantly increased risk of prostate cancer and/or are detected at significantly higher levels in prostate cancer patients than in controls. Also, more recently, a case-control study carried out in Guadeloupe has revealed that exposure to chlordecone, an organochlorine pesticide with strong oestrogenic properties used both in Martinique and Guadeloupe, is associated significantly with an increased prostate cancer risk [54].But this study does not prove that chlordecone, is the cause of the continuous growing incidence of prostate cancer in these two islands. Other factors including the use of other pesticide types may be involved. As suggested in Table 2, except for Sweden, for which factors other than pesticides are probably involved, the amount of pesticides used expressed per inhabitant appears to be more than four times higher in Martinique than what it is in metropolitan France, and there seems to be a statistically significant positive correlation between the incidence rates of prostate cancer and the levels of exposure to pesticides in the different countries analyzed, suggesting that among the environmental factors causally involved in prostate carcinogenesis the intensive use of pesticides could be implicated.

In conclusion, we suggest that the high incidence rate of prostate cancer in Martinique may, in fact, be the result of gene-environment interactions in highly genetically susceptible African descent individuals, that environmental factors may account for the continued increase of incidence of this cancer, and that among these factors, CMR or presumed CMR organochlorine pesticides may play a role. Further investigations are, however, needed to determine precisely which causative factors are actually specifically involved.

## Figures and Tables

**Table 1:** World age-standardized incidence rates (ASRs) of prostate cancer in 2005 in Caribbean, USA, UK, Sweden, metropolitan France, and Martinique. Comparison with percentages of African descents, life expectancy at birth, dietary energy and fat supply, and dietary energy and fat consumption.

Region	ASR 2005 specific registries^a^	ASR Globocan 2008^b^	African descents^c^ (%)	LEB^d^ (years)	DES (kcals)^e^	FS (g/person/day)^f^	DEC (Cal/person/day)^g^	FC (g/person/day)^h^
Caribbean								
Jamaica	—	*51.1 *	90.9	69	2 558	68	2 808	84
Cuba	**29.8 **	*53.8 *	11	78	3 129	83	3 275	54
Dominican Republic	—	*68.8 *	84	66	2 310	60	2 298	77
Haiti	—	*78.4 *	95	59	2 006	38	1 835	31
Bahamas	—	*78.5 *	85	71	2 776	91	2 690	93
Trinidad and Tobago	—	*89.4 *	39.5	66	2 770	71	2 759	77
Guadeloupe	—	*94.8 *	90	76	2 776	84	—	—
Puerto Rico	—	*102.2 *	—	—	—	—	—	—
Barbados	—	*140*	80	72	3 217	111	2 926	88
USA total	**106 **	*83.8 *	12.6	75	3 642	154	3 826	164
Black	164.8	—		—	—		—	—
White	101.8	—		—	—		—	—
UK	**52.2** ^ i^	* 62.1 *	2	77	3 270	142	3 426	137
Sweden	** 112.4**	* 114.2*	1.1	79	2 977	127	3 120	123
metropolitan France	** 121.2 **	* 118.3*	N/A	77	3 593	168	3 602	164

Martinique	** 177**	* 173.7*	80	76.5	2 768	84	** —**	**—**

^a^Age-standardized rates (ASR) are per 100 000 man-year and are age-adjusted to the IARC world standard population. Data source are obtained for Cuba from the Public Health Ministry [11], for USA, from the National Cancer Institute's Surveillance, Epidemiology and End Results (SEER) 2003–2007 [16], for UK, from the Information Services Division [12], Scotland [13] (see i), for Sweden, from the National Board of Health and Welfare [15], for metropolitan France, from the French National Sanitary Surveillance Institute (InVS) [9], and for Martinique, from AMREC [8].

^b^Data source for World ASR obtained from Globocan 2008 [10].

^c^Data source obtained from the Encyclopedia of the Nations [17], the Befolkningsstatistik [19], and the Office by National Statistics [18], for the Caribbean area, Sweden, and UK, respectively. The Caribbean people living in UK represent 1% of the overall population. For France, data are not available (N/A) for ethical considerations and legal regulation. Values are also supposed to be low, within the same range as what is estimated for Sweden and UK.

^d^Life expectancy at birth (LEB) (males). Data source obtained from the WHO Core Health Indicators for 2006 (WHO World health statistics, 2008).

^e^Dietary energy supply (DES), average total kilocalories available per person per day for the period 1988–1990. Data source obtained from FAO food balance sheets. National indices of dietary fat supplies [18, 22].

^f^Fat supplies (FS) are expressed as average grams of fat available per person per day for the period 1988–1990. Data source obtained from FAO food balance sheets. National indices of dietary fat supplies [18, 22].

^g^Dietary energy consumption (DEC) (Cal/person/day) for the period 2003–2005. Data source obtained from FAO Statistical Yearbook 2009 [19, 23].

^h^Fat consumption (FC) (g/person/day) for the period 2003–2005. Data source obtained from FAO Statistical Yearbook 2009 [19, 23].

^i^ASR 2005 determined from specific registries for the whole UK are not available. World ASR are 61.6 for England in 2002 and 52.2 for Scotland in 2005. Europe-ASR for England, Wales and Scotland in 2005 are 95.6, 112.9 and 79.6, respectively.

**Table 2:** Amounts of pesticides used in Martinique (in tons) in comparison with metropolitan France and other countries. Search for a correlation with the incidence rates of prostate cancer.

Region	Total amount^a,b^	Population^b^	Amount per inhabitant	ASR 2005^c^
Cuba	1 900	11 477 459	1·10^−4^	29.8
Sweden	1 553	9 074 055	1·10^−4^	112.4
UK	15 248	62 348 447	2·10^−4^	52.2
metropolitan France	89 084	63 136 180	1.4·10^−3^	121.2
USA	555 300	310 232 863	1.7·10^−3^	106
Martinique	2 500	414 516	6·10^−3^	177

^a^Amounts are expressed in tons.

^b^Values are indicated for 2000.

^c^Data from specific registries. See Table 1

**Table 3:** CMR and presumed CMR pesticides used in Martinique.

	On the market	Maximum of use	Withdrawal from the market for agricultural use	Continuation of use	IARC classification
Technical DDT	1939	1960–1990	1972	—	2B
Technical HCH	1940^a^	1950–1960	1988	1998	2B
Lindane	1940^a^	1950–1960	1992	—	2B
Aldrin/dieldrin	1950^a^	1960	1972	1992	3^b^
Chlordecone	1972	1980	1990	1993	2B
Chlordanes	1960^a^	—	—	—	2B
Simazine	1991^a^	—	2001	—	3^b^

^a^: Official data not available ^b^: Aldrin, Dieldrin, and Simazine although presently classified category 3 by IARC have been shown to be associated with an increased risk of prostate cancer (see text). Technical DDT is a mixture of the isomers p,p′-DDT (85%), o,p′-DDT (15%) and o,o′-DDT (<1%) and technical HCH, a mixture of the isomers *α*, *β*, and *γ*. Chlordanes include trans-chlordane, cis-chlordane, trans-nonachlor, cis-nonachlor, and heptachlor.

## Abbreviations

ASR: Age-standardized rate
CI: Confidence intervals
CMR: Carcinogenic, mutagenic, and/or reprotoxic
DDE: 1,1-dichloro-2,2^'^-bis-p-chlorophenyl-ethylene
DDT: Dichloro-diphenyl-trichloroethane
DEC: Dietary energy consumption
DES: Dietary energy supply
EPIC: European prospective investigation into cancer and nutrition
FC: Fat consumption
HCH: Hexachlorocyclohexane
IARC: International Agency for Research on Cancer
InVS: French National Sanitary Surveillance Institute
LEB: Life expectancy at birth
PSA: Prostate-specific antigen
REML: Restricted maximum likelihood
SEER: Surveillance, epidemiology, and end results.
